# Inhibition of Myocardial Ischemia/Reperfusion Injury by Exosomes Secreted from Mesenchymal Stem Cells

**DOI:** 10.1155/2016/4328362

**Published:** 2016-04-26

**Authors:** Heng Zhang, Meng Xiang, Dan Meng, Ning Sun, Sifeng Chen

**Affiliations:** Department of Physiology and Pathophysiology, Fudan University Shanghai Medical College, Shanghai 200032, China

## Abstract

Exosomes secreted by mesenchymal stem cells have shown great therapeutic potential in regenerative medicine. In this study, we performed meta-analysis to assess the clinical effectiveness of using exosomes in ischemia/reperfusion injury based on the reports published between January 2000 and September 2015 and indexed in the PUBMED and Web of Science databases. The effect of exosomes on heart function was evaluated according to the following parameters: the area at risk as a percentage of the left ventricle, infarct size as a percentage of the area at risk, infarct size as a percentage of the left ventricle, left ventricular ejection fraction, left ventricular fraction shortening, end-diastolic volume, and end-systolic volume. Our analysis indicated that the currently available evidence confirmed the therapeutic potential of mesenchymal stem cell-secreted exosomes in the improvement of heart function. However, further mechanistic studies, therapeutic safety, and clinical trials are required for optimization and validation of this approach to cardiac regeneration after ischemia/reperfusion injury.

## 1. Introduction

Given the limited regenerative captivity of heart cells, myocardial infarction has always been defined as irreversible disease, ultimately resulting in heart failure and death. Recently, it has been found that cardiac stem cells have a potential to differentiate into cardiomyocytes and vascular structures [1]. However, the number of these stem cells is too low to underwrite the restoration of severely impaired heart function. Several types of adult stem cells, including mesenchymal stem cells (MSCs), were used to treat myocardial infarction (MI) and produced optimistic results. It has been shown that the injection of MSCs derived from adult bone marrow into the area of infarction can reduce infarct size and restore heart function to some degree after ischemia/reperfusion injury [2, 3]. Although the approach is very promising, there are risks associated with MSC application for cardiac regeneration, such as immunoreactivity and oncogenicity [4]. Accumulating evidence suggests that some of MSC therapeutic effects can be reproduced by MSC-derived exosomes [5].

Exosomes are small membrane vesicles carrying DNA, RNAs, and proteins. They play important roles in cell-cell communication and interaction. The function of exosomes depends on cell origin of the exosomes. Mature dendritic cells (DCs) secrete exosomes that carry antigens which can induce antigen-specific immune responses in other DCs. Cho and colleagues [6] demonstrated that heat shock proteins-enriched exosomes can elicit antitumor responses in a MHC-independent manner. Exosomes released by melanoma cells prepare sentinel lymph nodes for tumor metastasis [7].

Stem cells are the most active and healthy cells. MSCs are a subset of adult stem cells that originate from the mesoderm. MSCs can easily be expanded ex vivo for a period of time without changing their characteristics. As described previously, MSCs have great potential in treating tissue injuries including myocardial infarction [2, 3], liver fibrosis [8], oxidative damage [9], and kidney injury [10]. MSCs of different tissue origin have similar therapeutic effects on myocardial injury [11–16]. The use of exosomes instead of the whole MSCs may circumvent the potential risks of MSC therapy. Therefore, therapeutic effects of MSC-derived exosomes on ischemia/reperfusion heart injury have been explored in a number of animal models. Although most of the studies have demonstrated the therapeutic potential of exosomes isolated from MSCs, the effects showed certain variability, which raises some concerns about the clinical application of this treatment approach.

In this paper, we performed meta-analysis of the preclinical data obtained in six original research studies to verify the quality and strength of existing evidence on the therapeutic potential of MSC-isolated exosome [11–16]. Furthermore, we attempt to provide an updated assessment of regenerative effects exhibited by exosomes in ischemia/reperfusion heart injury.

## 2. Methods

### 2.1. Literature Search

**Figure 1:**
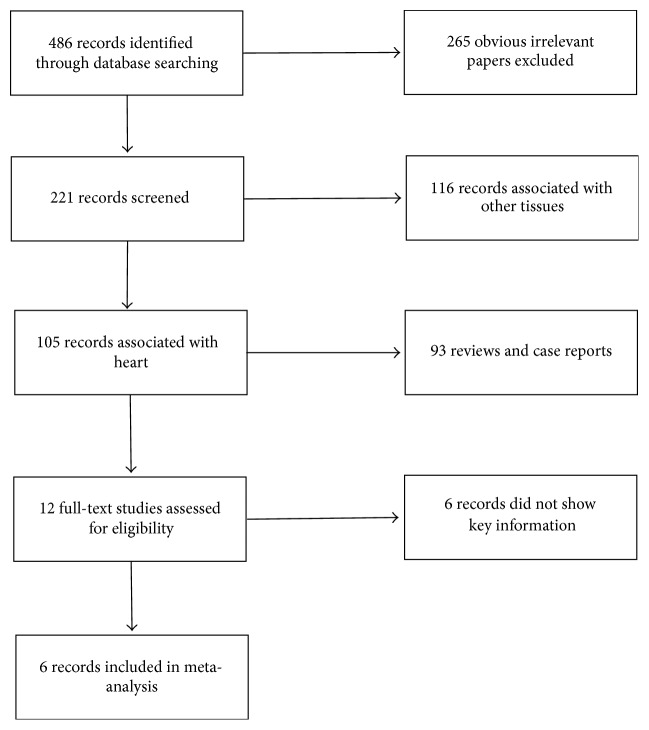
Results of systematic search of the literature.

Relevant papers published between January 2000 and September 2015 were screened in PUBMED and Web of Science databases using the following search terms: (“mesenchymal stem cell” OR “bone marrow stem cell”) AND (“exosomes” OR “conditioned medium (CM)”). Language was limited in English. The online search strategy used is shown in Figure 1.

### 2.2. Inclusion and Exclusion Criteria

Studies meeting the following criteria at the same time were included in this paper: (1) animal model: myocardial ischemia/reperfusion; (2) therapy: use of exosomes derived from MSCs; (3) detailed information of heart function and structure which was shown in the studies. Articles meeting any of the following criteria were excluded: case report, editorial, or letter to editors; other meta-analysis; studies with no precise data.

### 2.3. Validated Terms

The following general study information was extracted: last name of first author, year of publication, country, race of animal, MI and surgical procedure, the type of MSCs used to extract exosomes, and the amount of exosomes injected. Secondly, to evaluate the effects of exosomes on heart function, the following data was extracted: AAR as a percentage of the left ventricle (AAR/LV), infarct size as a percentage of the area at risk (IS/AAR), infarct size as a percentage of left ventricle (IS/LV), left ventricular ejection fraction (EF), left ventricular fraction shortening (FS), end-diastolic volume (EDV), and end-systolic volume (ESV).

### 2.4. Statistical Analysis

The meta-analysis of data was analyzed using Stata (version 12.0) and Engauge Digitizer (version 4.1) was used to extract the data of graph.

## 3. Results

### 3.1. Studies Included

After searching the databases, a total of six articles were identified to be proper for our analysis. The detailed information of articles was shown in Table 1. All articles chosen were published between 2008 and 2015, with two performed in the USA, two in Singapore, and two in Netherlands. Additional details not shown in Table 1 should be noted. Thus, in one study, animals were injected with CM twice (first 5 min prior to the onset of reperfusion and second immediately following reperfusion) [11], while in two studies, both HPLC-purified exosomes and CM were utilized in the experiments [12, 13]. In this analysis, we used only the data on purified exosomes. Other two studies compared the effect of the wild-type and genetically modified exosomes [15, 16]; however, we used only the data related to the wild-type exosomes to exclude the therapeutic effect of protein overexpression on MI. In one publication, we extracted the data on the effect of exosomes used at the concentration of 16 *μ*g/kg [14].

### 3.2. The Effectiveness of Exosomes on AAR/LV

**Figure 2:**
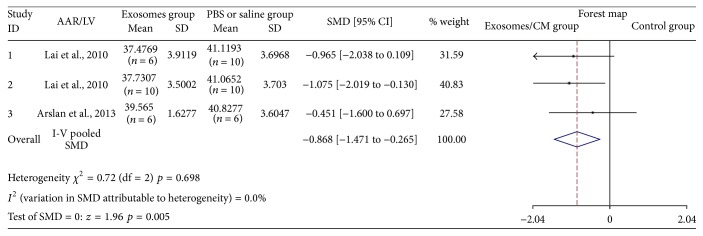
The effectiveness of exosomes on AAR/LV.

Three studies assessed AAR/LV after injection of exosomes [11, 12, 14]. Forty-eight animal cases (22 cases in experimental group; 26 cases in control group) were included. Generally, exosomes-based therapy was associated with mild AAR/LV reduction (SMD: −0.868, 95% CI: −1.471 to −0.265, *p* = 0.005); heterogeneity was not observed (*I* ^2^ = 0.0%, *p* = 0.698) (Figure 2). All articles showed similar AAR/LV decreasing trend in exosomes groups.

### 3.3. The Effectiveness of Exosomes on IS/AAR

**Figure 3:**
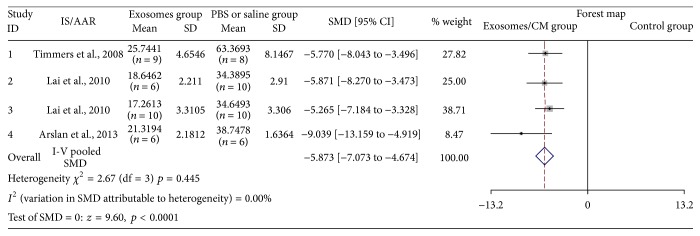
The effectiveness of exosomes on IS/AAR.

Four studies assessed IS/AAR after injection of exosomes [11–14]. Sixty-five animal cases (31 cases in experimental group; 34 cases in control group) were included. Generally, exosomes therapy was associated with significantly lower IS/AAR (SMD: −5.873, 95% CI: −7.073 to −4.674, *p* < 0.0001). Heterogeneity was observed (*I* ^2^ = 0.0%, *p* = 0.445) (Figure 3). All articles reported similar IS/AAR decreasing trend in exosomes groups.

### 3.4. The Effectiveness of Exosomes on IS/LV

**Figure 4:**
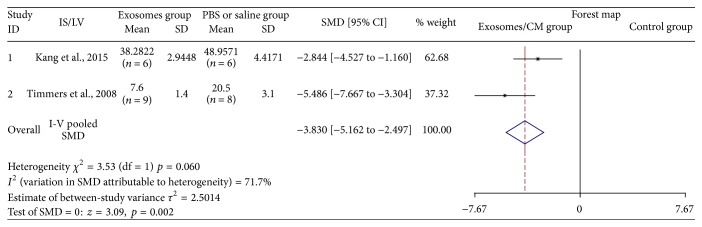
The effectiveness of exosomes on IS/LV.

Two studies assessed IS/LV after injection of exosomes [11, 15]. Twenty-nine animal cases (15 cases in experimental group; 14 cases in control group) were included. Generally, the effect of exosomes therapy was associated with IS/LV reduction (SMD: −3.830, 95% CI: −5.162 to −2.497, *p* = 0.002). Heterogeneity was not observed (*I* ^2^ = 71.7%, *p* = 0.06) (Figure 4). All articles showed similar trend in exosomes groups.

### 3.5. The Effectiveness of Exosomes on EF Group

**Figure 5:**
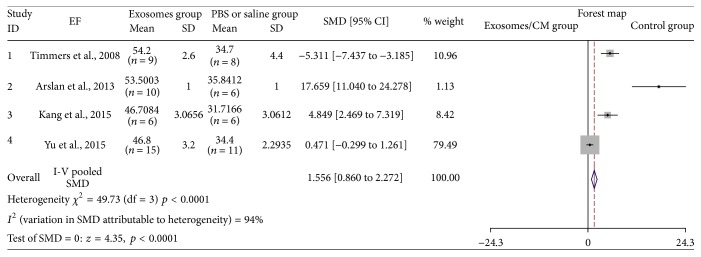
The effectiveness of exosomes on EF.

Four studies assessed EF after injection of exosomes [11, 14–16]. Seventy-one animal cases (40 cases in experimental group; 31 cases in control group) were included. Generally, the effect of exosomes therapy on upregulating EF was significant (SMD: 1.566, 95% CI: 0.860 to 1.261, *p* < 0.0001). However, certain heterogeneity was observed (*I* ^2^ = 94%, *p* < 0.0001) (Figure 5). While all studies demonstrated EF increase in the exosome-treated group, the increase of EF observed by Arslan et al. was significantly higher than others'. If this study was excluded, heterogeneity would decrease to an insignificant level.

### 3.6. The Effectiveness of Exosomes on FS

**Figure 6:**
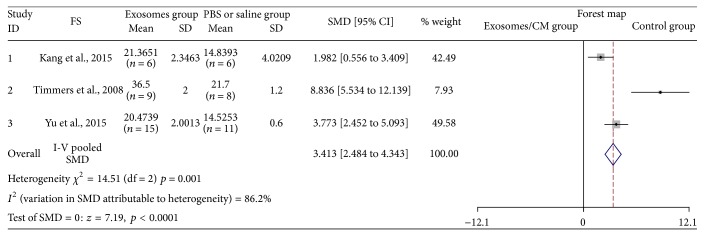
The effectiveness of exosomes on FS.

Three studies assessed FS after injection of exosomes [11, 15, 16]. Fifty-five animal cases (30 cases in experimental group; 25 cases in control group) were included. Generally, the effect of exosomes therapy on upregulating FS was significant (SMD: 3.413, 95% CI: 2.484 to 4.343, *p* < 0.0001) (Figure 6). Though all studies showed FS increase in the exosome-treated animals, the findings showed inconsistency, resulting in significant heterogeneity (*I* ^2^ = 86.2%, *p* = 0.001).

### 3.7. The Effectiveness of Exosomes on EDV

**Figure 7:**
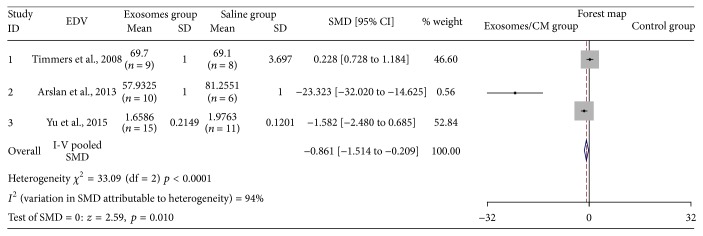
The effectiveness of exosomes on EDV.

Three studies assessed EDV after injection of exosomes [11, 14, 16]. Fifty-five animal cases (30 cases in experimental group; 25 cases in control group) were included. Generally, the effect of exosomes was associated with EDV reduction (SMD: −0.861, 95% CI: −1.514 to −0.209, *p* = 0.01) (Figure 7). However, Timmers et al. [11] have reported that EDV decrease by exosomes therapy was statistically insignificant, which resulted in heterogeneity among the analyzed data (*I* ^2^ = 94%, *p* < 0.0001).

### 3.8. The Effectiveness of Exosomes on ESV

**Figure 8:**
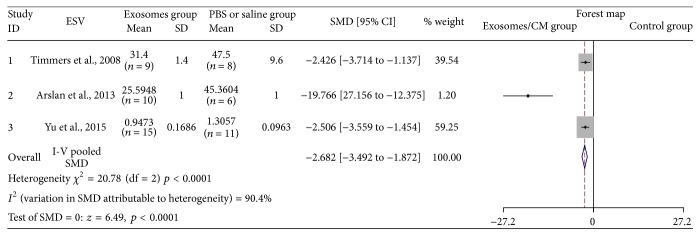
The effectiveness of exosomes on ESV.

Three studies assessed ESV after injection of exosomes [11, 14, 16]. Fifty-nine animal cases (34 cases in experimental group; 25 cases in control group) were included. Generally, the effect of exosomes therapy on lowering ESV was significant (SMD: −2.682, 95% CI: −3.492 to −1.872, *p* < 0.0001) (Figure 8); however, although ESV reduction trend in the exosomes-treated groups was consistent among the studies, considerable heterogeneity was observed (*I* ^2^ = 90.4%, *p* < 0.0001). Thus, the effect detected by Arslan et al. was markedly higher than that reported in the other two articles that demonstrated no heterogeneity.

## 4. Discussion

Myocardial infarction and associated complication are a great socioeconomic burden to healthcare system. Stem cells including MSCs therapy could be promising approach in curing patients with MI. Moreover, there is accumulating evidence that stem cell-secreted products could be used to treat MI-caused injuries [17, 18]. However, our search of the PUBMED and Web of Science was to find out that only one systematic review about the effects of microvesicle therapy on MI has been published in 2015 [19]. Although the systematic review included four articles related to therapy of exosomes or CM on MI injury, they reported only one clinical parameter (AAR), which is insufficient to make conclusions about the therapeutic potential of exosomes. In fact, there are 12 control trials published in 2008 to 2015 providing new evidence (unfortunately, six of them did not show the key information; we had to delete them). Thus, an updated meta-analysis is crucial. This meta-analysis is based on six controlled preclinical trials to demonstrate that exosomes or CM therapy could significantly improve heart function, in terms of IS/AAR, AAR/LV, IS/LV, EF, FS, EDV, and ESV.

In the mid-1980s, Johnstone [20] firstly reported that reticulocytes released the bulk of their transferrin receptor in association with small membrane vesicles. The exocytosed vesicles were uncovered after ultracentrifugation at 100,000 ×g for 90 min and were named exosome. Exosomes are bilipid membrane particles with a diameter of 50–100 nm. They carry various proteins and RNA to effect numerous pathways. Several studies reported that exosome was the key signal in cell-cell interaction. There is accumulating evidence that cells may transiently modify the phenotype of neighboring cells by proteins and RNAs through mechanism that involves exosomes [21]. Some of biological active components of MVs depend on the cell of origin. Microvesicle- (MV-) mediated exchange of receptors, proteins, mRNA, and miRNA between therapeutic cells and cells in damaged organs for MV delivering could temporally modify the phenotypes of these cells. Components of exosomes membrane, for example, sphingosine-1-phosphate (SIP), inhibit cell apoptosis and stimulate angiogenesis. The exosomes derived from MSCs have cardioprotective effects confirmed by some studies. Embryo stem cell-derived MVs can induce phosphorylation of MAPK p42/44 and serine-threonine kinase AKT [21]. Arslan et al. [14] demonstrated that exosomes can replenish depleted glycolytic enzymes to raise the level of ATP and NADH in the heart. Subsequently, they found out that exosomes derived from MSCs can manifestly increase AKT and GSK3 phosphorylation. Besides, proapoptotic phosphorylation of c-JNK was reduced. Based on these findings, the author concluded that exosomes can increase ATP levels and decrease oxidative stress and activated PI3K/Akt pathway to exert cardioprotective effects.

In IS/AAR group, Timmers et al. [11, 13, 14] all confirmed that hESE-MSC secretion reduces infarct size in mouse and pigs MI/R injury model. Though Timmers et al. used pigs as animal model, the meta-analysis showed positive results: IS/AAR was significantly decreased and heterogeneity was not observed, indicating that exosomes showed consistent therapeutic potential in reducing IS/AAR. Similar consistency was detected after the analysis of exosomes effects on AAR/LV and IS/LV [11–14].

However, in the parameters of heart function groups (EF, FS, EDV, and ESV), although heart function had been improved to some extent, the heterogeneity was observed and significant. For example, the data on ESV, EDV, and EF provided by Arslan et al. [14] was markedly higher than other studies' data. Arslan et al. [14] did not give the exact dose of exosome injected. Nor did they give the way of injection. This fact made us speculate that they may give more dose of exosomes or find a more effective way to inject the exosomes. On the other hand, the data on ESV and EDV provided by Yu et al. [16] was significantly lower than others' results. Yu et al. [16] did not give the precise dose of exosomes used either.

The current research has some limitations. First, the number of trials was relatively small. Second, the seven parameters we chose are not enough to show the whole function of exosomes. Third, the data of heart function parameters was not consistent causing the heterogeneity. Fourth, not all the articles gave the specific details we needed. Fifth, we acknowledge that the analysis may present a bias towards overestimation of positive outcomes, since negative results are less likely to be published. The relative homogeneity of this analysis is in certain contrast to the heterogeneity in clinical studies. Finally, we also should be reminded that none of the studies reviewed here mentioned potential serious adverse effects on other tissues; therefore, the safety of the exosome therapy is still unclear [19].

## 5. Conclusions 

Based on the published evidence, our meta-analysis presented a novel insight of the therapeutic effect of MSC-derived exosomes on MI. The preclinical results are encouraging for preparing and using feasibility studies in humans. We believed that further studies are required to validate the therapeutic safety in the future although the researchers did not find the side effect of exosomes derived from MSCs in animals. Validation of optimal conditions for their clinical application is also needed.

## Figures and Tables

**Table 1:** The detail information of articles included.

Study	Country	Animals	The source of MSCs	Particles	Purification method	Injury	Experimental group treatment	Control group treatment	Time point of extracting heart tissue	The time point of cardiac magnetic resonance imaging	Route
Timmers et al., 2008 [11]	Netherlands	Pig	hESC	10–220 nm	MW cutoff	LCA occlusion for 75 min	1 mL MSC-CM 4 mL MSC-CM^*∗*^	Saline	After 4 h of reperfusion	4 hours after reperfusion	Intracoronary injection

Lai et al., 2010 [13]	Singapore	Mouse	Fetal tissue	50–65 nm	HPLC	LCA occlusion for 30 min	10 *μ*g protein of HPLC F1	200 *μ*L saline	After 24 h of reperfusion	No	Via tail vein

Lai et al., 2010 [12]	Singapore	Mouse	HuES9.E1-derived	50–65 nm	HPLC	LCA occlusion for 30 min	0.4 *μ*g protein of HPLC F1	200 *μ*L saline	After 24 h of reperfusion	No	Via tail vein

Arslan et al., 2013 [14]	Netherlands	Mouse	HuES9.E1-derived	Not shown	MWCO	LCA occlusion for 30 min	0.4 *μ*g/mL exosomes	Not shown	After 24 h of reperfusion	1, 7, 28 days after LCA	Not shown

Kang et al., 2015 [15]	USA	Rat	Rat bone marrow	40–90 nm	ExoQuick-TC	LCA occlusion (time not shown)	Not shown	Not shown	Not shown	4 weeks after LCA	Not shown

Yu et al., 2015 [16]	USA	Rat	Rat bone marrow	100 nm	MWCO	LCA occlusion (time not shown)	Not shown	Saline	Not shown	4 weeks after LCA	Intramyocardial injection

^*∗*^Animals were injected with CM twice: 5 min prior to the onset of reperfusion and immediately following reperfusion, respectively.
